# Families in the COVID-19 pandemic: parental stress, parent mental health and the occurrence of adverse childhood experiences—results of a representative survey in Germany

**DOI:** 10.1007/s00787-021-01739-0

**Published:** 2021-03-01

**Authors:** Claudia Calvano, Lara Engelke, Jessica Di Bella, Jana Kindermann, Babette Renneberg, Sibylle M. Winter

**Affiliations:** 1 Department of Child and Adolescent Psychiatry, Psychosomatics and Psychotherapy, Charité – Universitätsmedizin Berlin, corporate member of Freie Universität Berlin, Humboldt-Universität zu Berlin, and Berlin Institute of Health, Augustenburger Platz 1, 13353 Berlin, Germany; 2 Department of Education and Psychology, Clinical Psychology and Psychotherapy, Freie Universität Berlin, Habelschwerdter Allee 45, 14195 Berlin, Germany

**Keywords:** COVID-19, Parental stress, Mental health, Adverse childhood experiences, Child maltreatment, Child abuse, Child neglect, Domestic violence

## Abstract

Parenting during the COVID-19 pandemic is highly challenging, with parents having to meet various demands simultaneously. An increase in adverse childhood experiences (ACEs) has been widely predicted, but empirical evidence is still scarce. This study aimed to (1) generate representative data on pandemic-related stress, parental stress, general stress, parental subjective and mental health, and the occurrence of ACEs; (2) identify risk factors for an increase in ACEs, and (3) provide qualitative data on parents’ experiences. A representative survey was conducted in Germany in August 2020 with 1024 parents of underage children (*M*_age_ = 41.70, 50.9% female). More than 50% of parents reported being stressed by social distancing and the closure of schools and childcare facilities. Parental stress increased significantly during the pandemic (*d* = 0.21). Subgroups of parents also reported very high levels of depressive symptoms (12.3%) and anxiety (9.7%). Up to one-third of the sample reported ACEs in the child’s lifetime. In this group, 29.1% reported an increase in children witnessing domestic violence during the pandemic, and 42.2% an increase verbal emotional abuse. These families were characterized by higher parental stress, job losses, and younger parent and child age. Positive aspects of the pandemic related primarily to personal or family life (e.g. slower pace of life, increase in family time). While some parents coped well, a particularly negative pattern was observed in a subgroup of families that experienced an increase in ACEs. Parental stress emerged as important target point for interventions addressing the negative sequelae of the pandemic.

**Supplementary Information:**

The online version contains supplementary material available at 10.1007/s00787-021-01739-0.

## Introduction

The SARS-CoV-2 coronavirus was declared a “public health emergency of international concern” by the World Health Organisation on January 30, 2020, and upgraded to a pandemic on March 11, 2020. In Germany, the first peak of infections occurred in spring 2020 and comprehensive, nationwide restrictions were implemented to slow down infection rates.

The restrictions are especially challenging for families, with home schooling, social distancing measures and lockdown situations having a profound and complex impact in the family context [1–3]. These parenting challenges are compounded by the demands of working from home, economic difficulties and layoffs, and social restrictions on parents [1, 2, 4]. It has been suggested that the profound changes to everyday family life caused by the pandemic may fuel parental stress and intrafamilial tension, which may in turn lead to an increase in adverse childhood experiences (ACEs), including domestic violence, child abuse, and neglect [5, 6].

While evidence on the psychosocial sequelae of the COVID-19 pandemic for general population samples [7] and for children and adolescents [8] is emerging, the situation of families and specifically parental burden has less frequently been analysed so far. To date, few studies have yet focused on pandemic-related stress, parental stress and the occurrence of ACEs during the pandemic: an online survey of 420 caregivers in the United States conducted in April 2020 showed a moderate level of general caregiver stress, mild generalized anxiety, and average depression [9]. The role of parental stress was also analysed in a study conducted in Italy with 824 parents of children aged 1–14 years in early April 2020, when the nationwide lockdown period was extended for the first time [10]. Poorer parental coping with the lockdown measures was related to higher individual parent stress, parenting stress and child behavioural problems. Although these findings underline the broad effects of the pandemic within the family context, the online sample was not representative.

The Canadian Perspectives Survey Series examined family stress and perceived risk for domestic violence due to the COVID-19 pandemic in 4627 adults [11]. The results suggest that financial insecurities due to changes in working conditions were the main cause of an increase in family stress and in the perceived risk for domestic violence. Reduced social contacts were also related to higher concerns about domestic violence. However, the actual occurrence of domestic violence was not assessed. An online survey with 258 parents living in Singapore found that parental stress mediated the association between the perceived impact of COVID-19 and harsh punishment of children [12]. However, the sample size was relatively low and the sample was recruited via Facebook and community organizations, limiting the representativeness of the study and restricting its generalizability beyond Singapore. Two recent studies add evidence on the assumed relation between pandemic-related stress and the occurrence of or risk for child abuse. Brown et al. [13] analysed 183 parents of underaged children, mainly mothers, and showed that pandemic-related stress, anxiety and depression are related to parents’ perceived stress levels. Furthermore, parents who were receiving financial assistance already pre-COVID and parents with higher anxiety and depression showed increased child abuse potential. The actual occurrence of psychological maltreatment and physical abuse was assessed in a recent study by Lawson et al. [14]. The authors analysed risk factors for child abuse among 342 parents of 4–10-year-old children in an online survey. The loss of job due to the pandemic, higher parental depression and previous emotional maltreatment were related to emotional maltreatment within the week prior to data collection. For the occurrence of physical abuse, loss of job emerged as relevant risk factor as well, however, the effect was weakened if parents showed higher positive cognitive reframing. While this study provided important data on risk and resilience factors for the occurrence of child abuse, the sample was recruited via social media channels, limiting representativeness. Furthermore, the role of parental stress and other pandemic-related stressors for the occurrence and increase of ACE during the pandemic has not been analysed so far.

By surveying a representative sample of parents with underage children, this study aims to (1) generate representative data on pandemic-related stress, parental stress, parental subjective and mental health and the occurrence of ACEs; (2) describe risk factors for an increase in ACEs and (3) provide qualitative data on parents’ negative and positive experiences during the pandemic.

## Methods

### Study design

The study population were German speaking households with underage children. Data were collected by the Berlin-based market research company INFO Marktforschungsinstitut using the survey software keyingress (Ingress GmbH). The survey was run between August 3rd and 11th 2020. We used a mixed mode design that combined computer-assisted telephone interviews (CATI; *n* = 402) with a computer-assisted web survey (CAWI; *n* = 622). For the recruitment of the CATI subsample, a dual-frame-design was used to include households with landline and parents with mobile phone numbers. Dual-frame design weighting accounted for different selection chances of parents available by landline vs. mobile phones and for the specific characteristics of landline users and mobile phone users. The telephone surveys were conducted by trained and supervised staff. Participants in the web survey were recruited from an active online-access panel. Participants received incentives from the panel provider to compensate their participation in the survey according to a fixed scheme. Respondents with an unrealistically short completion time (*n* = 66) were excluded from the CAWI data set. To provide representative data, the total sample was recruited according to current micro-census quota for the German population for age, sex, household size, educational level and residency.

### Population weighting

To increase representativeness and to limit bias, we applied post-stratification weighting for sociodemographic factors to account for disproportionalities between our recruited sample and the micro-census quota. The recruited sample was adjusted to the current micro-census quota in terms of parent age, parent gender, household size, parent educational level and residency. After an iterative weighting procedure, each case has received an individual weighting factor (rounded mean weight 1.000, rounded minimum weight 0.378, rounded maximum weight 3.648). In this paper, sample characteristics were presented both for the unweighted data and weighted data; statistical analyses were conducted with the weighted data set. The results and conclusion do not change when using the unweighted data.

### Measures

#### Sociodemographic data

Data were collected on parent age and sex, parental status (biological parent, step parent, other), marital status, number of children in the household, and children’s age and sex. Family socioeconomic status (SES) was classified as low, medium, or high on the Winkler Index according to German population-based reference data [15].

#### Parent-Related Risk Factors

In addition, parent-related risk factors for an increase in ACEs were assessed: the parent’s risk of alcohol abuse during the pandemic was assessed by the alcohol abuse module of the German version of the Patient Health Questionnaire (PHQ-D) [16, 17]; the presence of a mental disorder (“Have you ever been diagnosed with a mental illness by a physician?”), parent’s own history of child physical or sexual abuse (“In your childhood, have you been hit, punched or otherwise physically hurt during you childhood or adolescence, or have you been forced by someone to an unwanted sexual action during your childhood or adolescence?”), and the parent’s own experience of physical or sexual violence (“As an adult, have you been kicked, punched, or otherwise physically hurt, or have you been forced by someone to an unwanted sexual action?”) were assessed by self-report measures. Furthermore, parents were asked on the presence of a chronic or severe physical condition and whether they were among the risk group for COVID-19 [16, 18].

#### Pandemic-related experiences and stress

The Pandemic Stress Scale [19] was developed to assess COVID-19-related experiences and pandemic-related stress. COVID-19-related personal experiences were assessed in relation to the parents themselves or to other family or household members: By three items, contact to persons with a COVID-19-infection/hospital admission/death, was assessed. Furthermore, work-related data (short-time work, loss of job/work, severe financial loss) were collected. Furthermore, we asked the parents to indicate the month(s) with the subjectively highest burden by providing the months January 2020 till August 2020 separately, with the additional items “all months were equally stressful” and “no month was especially stressful”. For the assessment of pandemic-related stress, the parents rated the subjective burden of 13 restrictions (e.g. school closures) on a 5-point scale (anchors 1 = “not at all stressful”, 5 = “extremely stressful”). A higher sum score indicated a higher burden (Cronbach’s alpha = 0.94). Parents were asked to base their answers on the point at which they felt most stressed since the beginning of the pandemic. For a full presentation of this scale, please see Supplementary Material 1.

#### Parental stress

The Parental Stress Scale [20] is an 18-item self-report questionnaire on positive and negative perceptions of parenthood. Items are rated on a 5-point scale, with higher scores indicating higher parental stress. Parents were asked to rate their stress (1) at the time of the subjectively highest burden (Cronbach’s alpha = 0.88) and (2) in January 2020 (Cronbach’s alpha = 0.90).

#### General stress

The stress module of the PHQ-D [16] was used to assess general stress at the time of the subjectively highest burden. The module covers ten items on different psychosocial stressors, e.g., health concerns, concerns about weight or appearance, sexual problems or work-related stress (“How strongly did you feel impaired by the following problems?”). The items were answered on a three-point scale (0 = not impaired, 1 = a little impaired, 2 = strongly impaired). The sum score for general stress showed good internal consistency in our sample (Cronbach’s alpha = 0.81).

#### Parent mental health

The PHQ-4 [21], a four-item screening measure for generalized anxiety and depression, was used to measure parent mental health. Parents rated on a four-point scale how often they experienced symptoms (generalized anxiety: Cronbach’s alpha = 0.78, depression: alpha = 0.78, total score: alpha = 0.86) at the time of the subjectively highest burden.

#### Subjective health

We used a well-established single-item measure [22] to elicit parental self-ratings of health (“If you were to rate your general state of health on a scale from 0 to 10 (“0” meaning “couldn’t be worse” and “10” meaning “couldn’t be better”), how would you rate your current state of health?”). Subjective health was assessed (1) for the time of the highest burden and (2) for January 2020.

#### Adverse Childhood Experiences (ACEs)

To provide a comprehensive assessment of ACEs, we collected data on child abuse, neglect and household dysfunction [23, 24]. We adapted the items of the pediMACE [25–27]. Parents were asked to report the occurrence of ACEs for the children in their household. First, the occurrence of severe stressful life experiences (e.g. violence, abuse, neglect) was assessed, followed by ten items on specific subtypes of those events: five subtypes of child abuse (verbal emotional abuse towards the child, nonverbal emotional abuse towards the child, witnessing domestic violence, physical abuse, sexual abuse), three subtypes of neglect (emotional neglect, physical neglect, supervisory neglect), and two subtypes of household dysfunction (problems related to alcohol or substance use, mental illness in the household). Noteworthy, while the first item referred to severe forms of ACEs such as violence, abuse or neglect, item wordings of the subtypes mainly reflected low severity levels on the maltreatment classification system [23, 28]. Parents were first asked whether the distinct subtype of ACE had ever occurred in the child’s life and, if yes, to indicate the change in occurrence since the beginning of the pandemic on a five-point change scale (anchors “significantly more often” to “significantly less often”). For a full description of the items, see Supplementary Material 2.

#### Positive and negative experiences during the pandemic

In addition to the questionnaires, we asked two open questions on perceived highest burden and positive aspects of the pandemic: “Overall, what caused you the most stress during the pandemic?” and “What has changed for the better during the pandemic?”. Parents responded by typing their answers into a box (online survey) or by telling the interviewer (telephone survey).

### Sample characteristics

The sample comprised 1024 parents with a mean age of 41.7 years (SD = 8.37; range 18–73; weighted *M* = 40.89, SD = 8.17, range 18–73). Mean child age was 9.41 years (SD = 4.78, range 0.5–17.0; weighted *M* = 9.19, SD = 4.78, range 0.5–17.0). Table 1 summarizes the sociodemographic characteristics of the study participants, including both the unweighted raw data and the weighted data. In addition, comparison data from the recent German micro-census [29] are included in Table 1.

**Table 1:** Sociodemographic characteristics of the study participants

	Study sample *n* = 1024		Micro-census data^a^
	Unweighted	Weighted	
	*n* (%)	*n* (%)	%
Parent female	521 (50.9%)	534 (52.1%)	–
Biological parents	979 (95.6%)	979 (95.6%)	–
Single parents	116 (11.3%)	123 (12.1%)	–
Nationality			
German	1001 (97.8%)	1001 (97.8%)	92.3%
Other	23 (2.2%)	23 (2.2%)	7.7%
Number of children			
1 child	475 (46.4%)	474 (46.3%)	44.7%
2 children	422 (41.2%)	427 (41.7%)	37.5%
≥ 3 children	116 (11.3%)	122 (12.0%)	17.8%
Child age groups			
0–2 years	209 (20.4%)	230 (22.5%)	15.1%
3–5 years	247 (24.1%)	283 (27.6%)	15.4%
6–12 years	709 (69.2%)	713 (69.6%)	51.7%
13–17 years	537 (52.4%)	506 (49.4%)	17.7%
Marital status			
Married or in a relationship, same household	885 (86.4%)	874 (85.4%)	87.3%
Married or in a relationship, separate households	38 (3.7%)	43 (4.3%)	–
Not in a relationship or divorced	94 (9.2%)	100 (9.8%)	12.7%
Widowed	7 (0.7%)	6 (0.6%)	–
School education			
Low (up to 9 years of schooling)	83 (8.1%)	109 (10.7%)	20.0%
Middle (10 years of schooling)	365 (35.6%)	480 (46.8%)	33.2%
High (up to 13 years of schooling)	470 (45.9%)	426 (41.6%)	42.6%
No school education, other, missing data	6 (0.6%)	8 (0.8%)	4.3%
Current employment status			
Not employed (e.g. retired)	62 (6.1%)	65 (6.3%)	–
Unemployed	34 (3.3%)	40 (3.9%)	–
Furloughed	51 (5.0%)	56 (5.4%)	–
In part-time employment	276 (27.0%)	282 (27.6%)	29.2%
In full-time employment	590 (57.6%)	570 (55.7%)	70.8%
In training or student	11 (1.1%)	10 (1.0%)	–
Socioeconomic status index^b^			
Low	75 (7.3%)	101 (9.9%)	20.2%^c^
Middle	555 (54.2%)	589 (57.5%)	59.7%^c^
High	384 (37.5%)	332 (32.4%)	20.1%^c^

^a^Population-based comparison data derived from the Micro-Census for Germany for 2019, for 2018 (school education) and for 2011 (nationality, child age groups) [29]

^b^Index calculated according to the Winkler Index [15]

^c^Population-based reference data (*n* = 12.292) for socioeconomic index derived from [15]

Data on COVID-19-related experiences and parent-related risk factors are summarized in Table 2. Almost half of the sample identified April and May 2020 as the most stressful months. After that, a continuous decrease was observed until August 2020.

**Table 2:** COVID-19-related experiences and parent-related risk factors: descriptive data (N = 1024)

	Unweighted	Weighted
	*n* (%)	*n* (%)
COVID-19-related experiences		
Effects of the pandemic on health situation		
Family/household member infected with COVID-19	23 (2.2%)	22 (2.2%)
Family/household member admitted to hospital with COVID-19	7 (0.7%)	8 (0.7%)
Family/household member died with COVID-19	4 (0.4%)	4 (0.4%)
Parent belongs to risk group for severe COVID-19^a^	96 (9.4%)	103 (10.1%)
Effects of the pandemic on job situation		
Reduced working hours	270 (26.5%)	277 (27.0%)
Job loss	54 (5.3%)	55 (5.4%)
Significant financial loss	212 (20.7%)	221 (21.5%)
Most stressful month		
February	16 (1.6%)	21 (2.0%)
March	266 (26.0%)	268 (26.1%)
April	500 (48.8%)	487 (47.5%)
May	426 (41.6%)	430 (42.0%)
June	252 (24.6%)	263 (25.7%)
July	112 (10.9%)	117 (11.4%)
August	45 (4.4%)	46 (4.5%)
All months equally stressful	117 (11.4%)	125 (12.2%)
No month was especially stressful	186 (18.2%)	186 (18.2%)
Parent-related risk factors		
Parental risk of alcohol abuse^b^	55 (5.4%)	56 (5.5%)
Parental mental disorder	95 (9.3%)	107 (10.4%)
Parental history of child abuse or neglect	222 (21.7%)	238 (23.2%)
Parental experience of violence in adulthood	108 (10.5%)	114 (11.2%)

^a^*n* = 141 (weighted: *n* = 146) parents reported a chronic or severe health condition [18]

^b^*n* = 727 (weighted: *n* = 700) parents indicated that they regularly drink alcohol, of these, *n* = 55 (weighted: *n* = 56) were at risk for alcohol abuse, according to PHQ-D[16]

### Data analysis

For data analysis and inferential statistics, the weighted data set was used. Outcome measures were analysed by descriptive statistics and compared with reference scores by *t* tests. Answers to the open questions were analysed by means of content analysis. Based on the first 100 answers, the team developed categories for negative and positive experiences in mutual discourse. Each answer could be classified to multiple categories. Two members of the team coded all answers independently. Discrepancies were discussed in the team until a final decision was reached. Frequencies of each category were calculated.

## Results

**Fig. 1:**
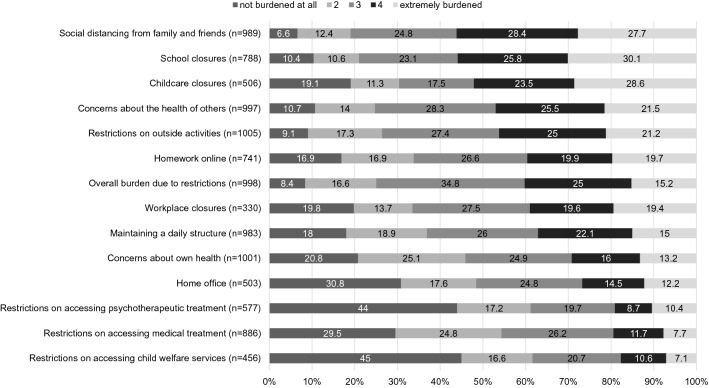
Stress due to pandemic-related restrictions, ordered by highest stress (score ≥ 4). Items rated as “not applicable” were excluded from the analysis, resulting in varying subsample sizes

First, we analyzed data on pandemic-related stress and the other outcome measures. The level of pandemic-related stress varied across domains (see Fig. 1). Parents felt most stressed (ratings 4 or 5 on the Pandemic Stress Scale) by social distancing from family and friends (56.1%), closure of schools (55.9%), closure of childcare (52.1%), concerns about the health of others (47.0%), and restrictions on outside activities (46.2%). The total sum score of pandemic-related stress was *M* = 31.97 (SD = 10.96; range 1–70).

Parental stress at the time of the subjectively highest burden was significantly higher than pre-COVID-19 levels (*M* = 36.93, SD = 10.45, range 18–71 vs. pre-COVID-19 *M* = 34.72, SD = 10.63, range = 18–70; *t*(1023) = 12.474, *p* < 0.001); the effect size was small (*d* = 0.21). However, parental stress at the time of the subjectively highest burden was not higher than in two reference samples (US sample [20]: *n* = 116, *M* = 37.1, SD = 8.1; German sample [30]: *n* = 121, *M* = 37.18, SD = 7.70) and was significantly lower than in clinical reference samples [31] (parents in treatment for their child’s behaviour problems: *n* = 51, *M* = 43.2, SD = 9.1; parents in inpatient psychiatric treatment: *n* = 83, *M* = 41.9, SD = 9.4).

The mean score for parents’ general stress at the time of the subjectively highest burden was *M* = 5.28 (SD = 4.13, range 0–20) and thus in the low range [16].

Parents rated their overall health status at the time of the subjectively highest burden as significantly worse than pre-COVID-19 levels (*M* = 6.80, SD = 2.21 vs. pre-COVID-19 *M* = 7.34, SD = 2.04; *t*(1023) = 10.33, *p* < 0.001). The effect size was small (*d* = 0.31).

**Fig. 2:**
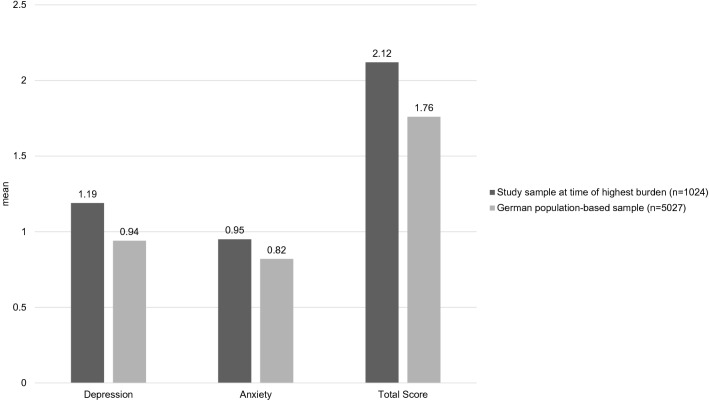
Comparison of PHQ-4 scores for depression, anxiety, and total score in our sample and in German normative data [21]. All between-group differences were significant at *p* < 0.001

Regarding parent mental health, our sample reported significantly higher symptoms of anxiety and depression than the German normative data [21] (see Fig. 2); the effect sizes were small (depression *d* = 0.21; anxiety *d* = 0.12, total score *d* = 0.18). 12.3% of the sample scored above the 95th percentile of the PHQ-4 [21] for symptom levels for depression; 9.7% for anxiety; 7.4% for the total score.

As women usually report higher levels of subjective distress than men, we checked for gender differences. Mothers scored higher than fathers on general stress (*d* = 0.32, *p* < 0.001), anxiety (*d* = 0.28, *p* < 0.001), and depression (*d* = 0.28, *p* < 0.001) and showed poorer subjective health (*d* = 0.15, *p* = 0.018). No sex differences emerged for pandemic-related stress (*d* = 0.09, *p* = 0.160) and parental stress (*d* = 0.08, *p* = 0.196).

Pandemic-related stress and parental outcomes were significantly correlated, with moderate effect sizes (see Table 3).

**Table 3:** Correlation between pandemic-related stress and parental outcomes^a^

		2	3	4	5	6
1	Pandemic-related stress	0.342	0.417	− 0.257	0.304	0.295
2	Parental stress		0.425	− 0.351	0.363	0.412
3	General stress			− 0.474	0.478	0.529
4	Subjective health				− 0.394	− 0.422
5	Anxiety					0.618
6	Depression					–

^a^All parental outcomes with reference to the time of the subjectively highest burden. For subjective health, higher scores indicate better health; for the other outcomes, higher scores indicate higher stress and symptoms. All correlations were significant with *p* < 0.001

To gain a more detailed picture of the associations between pandemic-related stress and parental outcomes, we conducted an item-wise analysis of the Pandemic Stress Scale (see Supplementary Material 3). Results showed a homogenous pattern: all outcomes were significantly related to the specific restrictions, with two exceptions (correlations subjective health with child care closures *r* = 0.002, and with job closures *r* = − 0.102). Coefficients of significant correlations were in the small to medium range (*r* = − 0.077 to *r* = 0.393).

### Occurrence of ACEs during the pandemic

**Fig. 3:**
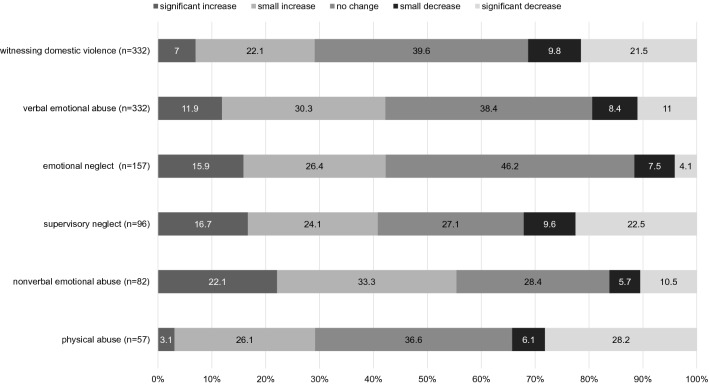
Change in occurrence of the subtypes of child abuse and neglect during the pandemic relative to pre-COVID-19 levels. The *n* in brackets indicates the number of parents reporting lifetime occurrence

6.5% (*n* = 66) of parents reported on their children’s lifetime occurrence of severe stressful life experiences including violence, abuse, or neglect. Of these, 34.8% reported an increase in occurrence during the pandemic (17.6% no change, 47.5% decrease). Concerning the specific subtypes of ACE, rates of lifetime occurrence were mainly higher. The highest lifetime occurrence was reported for children witnessing domestic violence (*n* = 332, 32.4%) and for verbal emotional abuse against the children (*n* = 332, 32.4%). Figure 3 displays the results for change in occurrence of the subtypes of child abuse and neglect during the pandemic relative to pre-COVID-19 levels. There were few reports of sexual abuse (*n* = 14) and physical neglect (*n* = 11). These subtypes were therefore excluded from further analyses, as conclusions would be limited. Note that across the subtypes, 27.1–46.2% of cases reported no change in occurrence and that in 11.6 − 34.3% of cases, a decrease was reported.

In terms of the subtypes of household disfunction, 143 parents (13.9%) reported that their child(ren) had been exposed to mental illness in the household in their lifetime; of these, 10.9% reported that domestic problems related to the mental illness had significantly increased during the pandemic (17.8% small increase, 42.4% no change, 2.2% small decrease, 26.7% significant decrease). 37 parents (3.7%) reported alcohol or substance abuse in the household over the child(ren)’s lifetime; in 5.1% of these cases, problems significantly increased during the pandemic (11.3% small increase, 45.9% no change, 2.0% small decrease, 35.7% significant decrease).

### Factors associated with an increase in ACEs

**Fig. 4:**
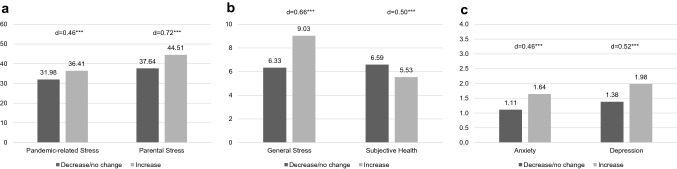
Comparison of outcome measures in cases where frequency of witnessing domestic violence increased vs. decreased/did not change during the pandemic for **a** pandemic-related stress and parental stress, **b** general stress and subjective health, and **c** anxiety and depression. Cohen’s d for effect size (*d* = 0.20: small, *d* = 0.50: medium, *d* = 0.80 large); *p < .05, **p < 0.01, ***p < 0.001

**Fig. 5:**
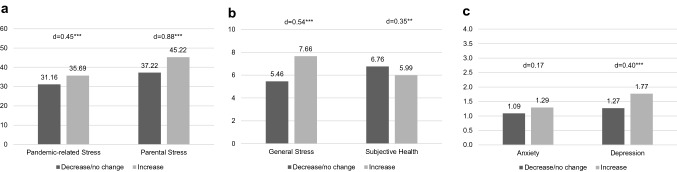
Comparison of outcome measures in cases where frequency of verbal emotional abuse increased vs. decreased/did not change during the pandemic for **a** pandemic-related stress and parental stress, **b** general stress and subjective health, and **c** anxiety and depression. Cohen’s *d* for effect size (*d* = 0.20: small, *d* = 0.50: medium, *d* = 0.80: large); *p < 0.05, **p < 0.01, ***p < 0.001

We examined the relationships between the parental outcomes and an increase in the two ACEs with the highest lifetime occurrence: witnessing domestic violence and verbal emotional abuse. Results are shown in Figs. 4 and 5, respectively. Parents reporting an increase in an ACE also reported higher pandemic-related stress and poorer parental outcomes, with small to medium effect sizes for all measures. The largest effect sizes were observed for parental stress. This pattern was also observed for the other ACE subtypes (see Supplementary Material 4 for a detailed summary of mean scores and effect sizes for all ACEs).

With respect to sociodemographic characteristics, analyses showed that the parents and children in the subgroups reporting a pandemic-related increase in witnessing domestic violence (WDV) or verbal emotional abuse (VEA) were significantly younger (see Supplementary Material 5). Concerning pandemic-specific factors, job losses and financial losses were related to an increase in WDV and VEA: 48.4% of the families with job losses during the pandemic reported an increase in WDV (vs. 27.2% of the families without job losses, *p* = 0.013), and 62.1% reported an increase in VEA (vs. 40.5% of the families without job losses, *p* = 0.024). In families reporting significant financial losses during the pandemic, 53.0% reported an increase in VEA (vs. 38.6% in families without financial loss, *p* = 0.021); the differences with respect to WDV were not statistically significant.

Concerning parent-related risk factors, 37.8% of parents with a history of child physical or sexual abuse reported an increase in WDV (vs. 24.9% of parents with no history of child physical or sexual abuse, *p* = 0.014). 61.5% of parents reporting the experience of physical or sexual violence in adulthood indicated an increase in VEA (vs. 38.7% of parents with no experience of physical or sexual violence in adulthood, *p* = 0.002). The other risk factors were not significantly related to an increase in the ACEs (see Supplementary Material 5).

### Qualitative data on perceived highest burden and positive aspects of the pandemic

**Fig. 6:**
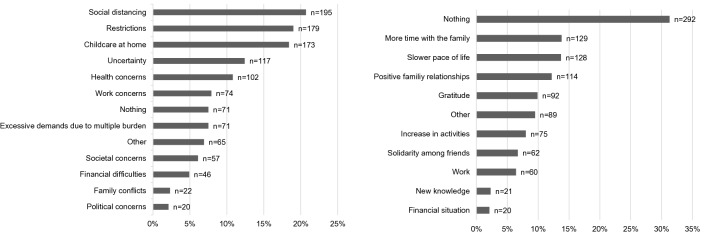
**a** Factors that burdened parents most during the pandemic. Data from 941 participants. Multiple categories per case (*M*_categories_ = 1.27). **b** Positive aspects of the pandemic. Data from 941 participants. Multiple categories per case (*M*_categories_ = 1.16)

A total of 941 participants (*N*_answers_ = 1192) provided answers to the open question asking what had caused them most stress during the pandemic (see Fig. 6a). The most frequent categories were social distancing (e.g. limitation of contacts, loneliness), restrictions (e.g. on leisure, healthcare, shopping; wearing a mask) and childcare at home. The responses reflect the breadth of stressful experiences during the pandemic, covering uncertainty about the course and consequences of the pandemic (e.g. “Nothing was concrete, clear and foreseeable”) as well as societal, financial, occupational and family aspects. The qualitative results validate and add to the quantitative findings of the Pandemic Stress Scale.

A total of 932 participants (*N*_answers_ = 1082) answered the question on the positive aspects of the pandemic (see Fig. 6b). The most frequent response (*n* = 292) was “nothing”, indicating that participants had difficulty seeing any kind of silver lining. Most responses related to positive outcomes for the family and the parents themselves, e.g., slower pace of life (e.g., “I’m calmer, more relaxed; I’m better able to deal with everything”), gratitude, and an increase in family time.

## Discussion

This study is among the first to systematically analyse the effects of the corona pandemic on parental health and stress and to examine whether it was indeed related to an increase in the occurrence of ACEs. Overall, the negative effects on parental outcomes were small to moderate. While parental stress increased during the pandemic, it was still within the average range; subjective health decreased moderately; rates of anxiety and depressive symptoms increased, with small effects. Pandemic-related stress varied across domains; parents felt most stressed by social distancing measures and by the closure of schools and childcare facilities. Higher pandemic-related stress was related to poorer parental outcomes and an increase in ACEs. Furthermore, subgroups with an increase in ACEs were characterized by specifically high pandemic-related stress and poor parental outcomes.

Severe ACEs were reported by 6.5% of the sample, which is in line with current prevalence rates [32]. For the specific subtypes, covering less severe forms of ACEs, a lifetime prevalence of up to 32% was reported, also in line with the literature [32]. In the subgroup of parents who reported lifetime ACEs, between 16.4 and 55.4% reported an increase since the beginning of the pandemic. In these families, poorer outcomes were observed on all measures (pandemic-related stress, parental stress, general stress, subjective health, anxiety and depression) than in families reporting no change or a decrease. This adds to findings on the relation between parental anxiety and depression and risk for child abuse [13]. Especially, and consistently across categories, parental stress was higher in families where ACEs increased. Noteworthy, parents in this subgroup also reported higher pre-pandemic levels of parental stress than the families with a decrease or no change in ACEs (WDV *d* = 0.50, VEA *d* = 0.45). Including the score for pre-pandemic parental stress as covariate in the analyses did not change the results. Other studies conducted during the COVID-19 pandemic have also highlighted the relevance of parental stress for child-related outcomes, such as child behavior problems [10] and parent–child closeness [12]. Parental stress thus seems to be a central variable reflecting the impact of the pandemic on families—both on the parents themselves and on their children.

Parental stress was not the only factor related to an increase in the two most prevalent ACEs: witnessing domestic violence and verbal emotional abuse. Families with younger children were especially at risk, which may reflect the impact of childcare and school closures [33]. Contrary to our expectations, single parenting, parent sex, belonging to the COVID-19 risk group, risk of alcohol abuse, and low SES were not related to an increase in ACEs. Although low case numbers limit the ability to draw conclusions and may explain contradictions with other recent findings [13, 34], our data suggest that negative effects on parent or child level may be attributable less to general sociodemographic or socioeconomic factors and more to pandemic-specific socioeconomic factors, such as job losses and financial difficulties. This finding is in line with Beland et al. [11], who reported an association between financial worries, domestic violence and family stress for a population-based sample, and with Lawson et al. [14], who identified pandemic-related job loss as a risk factor for the occurrence of emotional maltreatment and physical abuse. In line with the literature [35], a parent’s own history of violence was related to an increase in ACEs, although the effects emerging were mixed. Taken together, our results suggest that beyond younger age and parent’s own history of violence, job losses and financial difficulties additionally contributed to an increase in ACEs during the pandemic. The results of our study underline that the effects of the pandemic are heterogeneous and the stress imposed by the pandemic might especially exacerbate in known risk groups, which corresponds to other studies (see also [12, 14, 34]). However, longitudinal data are needed to confirm these findings.

Parental stress and general stress were not much higher than usual in our sample, in line with other recent findings [9, 36]. However, in contrast to other studies [13], levels of anxiety and depression were also comparatively low, with only 7–12% of respondents scoring above the 95^th^ percentile of the PHQ-4. A recent meta-analysis of 17 studies conducted mostly in Asia found a much more pronounced increase in rates of anxiety and depression, of up to 18.7–50.9% [7]. These differences may be explained by the phase of the pandemic in which the data were collected [5]. Most studies published thus far were conducted during earlier, more acute stages of the pandemic; our study was conducted in August 2020, when the first peak of the pandemic was over in Germany, the lockdown measures had been relaxed, and many regions were on school holidays. This situation may also have biased parents’ ratings with respect to the subjectively hardest time, as recent data suggest a decline in anxiety and depression over the first 20 weeks after implementation of lockdown measures [37]. Furthermore, up to August 2020, the situation in Germany was more stable than e.g., in other European countries [38], which may also explain cross-national differences.

The qualitative data on the aspects that caused parents most stress validated and added to the quantitative data from the Pandemic Stress Scale. Participants reported being particularly stressed by social distancing, restrictions and childcare at home. About one-third of participants could not identify any positive aspects of the pandemic; however, parents perceived more time for themselves and for the family and an improvement in family cohesion as positive aspects of the pandemic [5, 39].

In sum, parental stress emerged as important target point for interventions. The qualitative data also identified promising areas for resource-oriented interventions to mitigate the detrimental effects of the pandemic [39]. Prospective studies on the mediating role of parental stress and interactions with potential risk and resilience factors for parent and child outcomes are needed to provide insights into the pathways between these variables, as first evidence suggests [14].

One strength of this study is the mixed mode approach to data collection, reducing the bias associated with collecting data online only. Unlike other parent samples, in which mothers tend to be heavily overrepresented, our sample included equal proportions of mothers and fathers. Mothers reported higher general stress, anxiety and depression, in line with both the literature in general [21] and data collected during the pandemic [34, 37]. Notably, mothers and fathers did not differ in the level of pandemic-related stress and parental stress reported. While this finding is in line with data reported in a validation study of the Parental Stress Scale [20], pandemic-specific comparison data are as yet scarce. While we aimed to yield a representative sample in terms of parental education, we observed an imbalance compared to population-based reference data for parental education and SES index [15, 29]. The share of low SES in our sample was smaller than expected [15]. This might be attributable to the requirement of German language skills for participation and the low share of migrant families within the sample [40]. Therefore, generalization of the results to families with migrant background, low education, low SES or other socioeconomic risks is limited.

Concerning the assessment of ACEs, the parent-report on lifetime occurrence of ACEs provided plausible data, as with 6.5% for severe experiences up to 32% for the less severe forms, the data correspond to prevalence estimations in the general population [32]. Regarding the change in the occurrence of ACEs during the pandemic, we can only draw conclusions for the parents affirming lifetime occurrence. Furthermore, we do not have information about the absolute frequency in occurrence. It should be noted that the wording of the items used to assess the ACE subtypes mainly reflected low severity levels on established classification systems [23, 28]. The items included only few sample situations for the specific subtypes and the broad range of adverse experiences within the subtypes was not covered. Consequently, the data cannot be generalized to more severe cases of these subtypes. Overall, 11.6–37.7% of the sample reported a decrease in ACEs and 27.1–46.2% reported no change, suggesting that a significant number of families may be less affected or even relieved during the pandemic. Further analyses on parental resources and predictors of positive outcomes during the pandemic are warranted.

For reasons of parsimony, we did not assess pre-COVID-19 data on all measures, which prohibited analyses of change in general stress and mental health. Furthermore, pre-COVID-19 data were assessed retrospectively and we only have parent’s self-reported data and child-related outcome measures were not included in the study. Given the cross-sectional design of the survey, the results are solely correlational in nature and causal inferences cannot be drawn. Prospective studies are needed to replicate the predictive role of the risk factors for an increase in witnessing domestic violence and verbal emotional abuse, as well as the interplay between the predictors.

The results of this study confirm that the pandemic has had a profound impact on families. As school closures and social distancing from family and friends were among the most burdening restrictions for parents, great caution is necessary in applying these measures. Furthermore, additional efforts need to be put into helping families deal with the restrictions and “the new normal” [5]. Structural interventions targeting parents’ working conditions, such as offering parental leave for one parent, might provide relief during phases of lockdown. Targeted low-threshold online interventions aiming to activate parents’ intra- and inter-personal resources are warranted. Support for dealing with specific lockdown measures, such as not only help with home schooling, but also addressing parents’ own needs and their role as parents, may be a promising approach to specifically address parental stress and mental health. Child welfare services need to be available and should actively explore the presence of ACEs, including child abuse and neglect to provide targeted interventions [2]. Our findings from a population-based sample indicate that rates of child abuse and neglect increased in about one-third during the pandemic. Further research on high risk groups, in socioeconomic and psychosocial terms, is urgently needed.

## Supplementary Information

Below is the link to the electronic supplementary material.

Supplementary file1 (PDF 174 KB)

Supplementary file2 (PDF 107 KB)

Supplementary file3 (PDF 102 KB)

Supplementary file4 (PDF 125 KB)

Supplementary file5 (PDF 121 KB)

## Data Availability

Individual participant data that underlie the results reported in this article, after deidentification, will be made available to researchers who provide a methodologically sound proposal. Proposals should be directed to claudia.calvano@charite.de; to gain access, data requestors will need to sign a data access agreement.

## References

[1] Cluver L, Lachman JM, Sherr L, Wessels I, Krug E, Rakotomalala S, Blight S, Hillis S, Bachman G, Green O, Butchart A, Tomlinson M, Ward CL, Doubt J, McDonald K (2020). Parenting in a time of COVID-19. Lancet.

[2] Clemens V, Deschamps P, Fegert JM, Anagnostopoulos D, Bailey S, Doyle M, Eliez S, Hansen AS, Hebebrand J, Hillegers M, Jacobs B, Karwautz A, Kiss E, Kotsis K, Kumperscak HG, Pejovic-Milovancevic M, Christensen AMR, Raynaud J-P, Westerinen H, Visnapuu-Bernadt P (2020). Potential effects of “social” distancing measures and school lockdown on child and adolescent mental health. Eur Child Adolesc Psychiatry.

[3] Bruining H, Bartels M, Polderman TJC, Popma A (2020). COVID-19 and child and adolescent psychiatry: an unexpected blessing for part of our population?. Eur Child Adolesc Psychiatry.

[4] Gallagher S, Wetherell M (2020). Risk of depression in family caregivers: Unintended consequence of COVID-19. medRxiv.

[5] Fegert JM, Vitiello B, Plener PL, Clemens V (2020). Challenges and burden of the Coronavirus 2019 (COVID-19) pandemic for child and adolescent mental health: a narrative review to highlight clinical and research needs in the acute phase and the long return to normality. Child Adolesc Psychiatry Ment Health.

[6] Mahase E (2020). Covid-19: EU states report 60% rise in emergency calls about domestic violence. BMJ.

[7] Salari N, Hosseinian-Far A, Jalali R, Vaisi-Raygani A, Rasoulpoor S, Mohammadi M, Rasoulpoor S, Khaledi-Paveh B (2020). Prevalence of stress, anxiety, depression among the general population during the COVID-19 pandemic: a systematic review and meta-analysis. Globalization and Health.

[8] Stavridou A, Stergiopoulou AA, Panagouli E, Mesiris G, Thirios A, Mougiakos T, Troupis T, Psaltopoulou T, Tsolia M, Sergentanis TN, Tsitsika A (2020). Psychosocial consequences of COVID-19 in children, adolescents and young adults: a systematic review. Psychiatry Clin Neurosci.

[9] Russell BS, Hutchison M, Tambling R, Tomkunas AJ, Horton AL (2020). Initial challenges of caregiving during COVID-19: caregiver burden, mental health, and the parent-child relationship. Child Psychiatry Hum Dev.

[10] Spinelli M, Lionetti F, Pastore M, Fasolo M (2020). Parents' stress and children's psychological problems in families facing the COVID-19 outbreak in Italy. Front Psychol.

[11] Beland L-PB, Abel S, Haddad J, Mikola D (2020) Covid-19, family stress and domestic violence: remote work, isolation and bargaining power. In: GLO Discussion Paper. Essen

[12] Chung G, Lanier P, Ju PWY (2020). Mediating effects of parental stress on harsh parenting and parent-child relationship during Coronavirus (COVID-19) pandemic in Singapore. J Fam Violence.

[13] Brown SM, Doom JR, Lechuga-Peña S, Watamura SE, Koppels T (2020). Stress and parenting during the global COVID-19 pandemic. Child Abuse Negl.

[14] Lawson M, Piel MH, Simon M (2020). Child maltreatment during the COVID-19 pandemic: consequences of parental job loss on psychological and physical abuse towards children. Child Abuse Negl.

[15] Lampert T, Müters S, Stolzenberg H, Kroll LE, Group KS (2014) Messung des sozioökonomischen Status in der KiGGS-Studie. Bundesgesundheitsblatt-Gesundheitsforschung-Gesundheitsschutz 57(7):762–770 10.1007/s00103-014-1974-8 24950825

[16] Löwe B, Spitzer RL, Zipfel S, Herzog W (2002) PHQ-D: Gesundheitsfragebogen für Patienten. Manual Komplettversion und Kurzform. Universität Heidelberg, Heidelberg

[17] Finlay I, Gilmore I (2020). Covid-19 and alcohol—a dangerous cocktail. BMJ.

[18] Clark A, Jit M, Warren-Gash C, Guthrie B, Wang HHX, Mercer SW, Sanderson C, McKee M, Troeger C, Ong KL, Checchi F, Perel P, Joseph S, Gibbs HP, Banerjee A, Eggo RM, Nightingale ES, O'Reilly K, Jombart T, Edmunds WJ, Rosello A, Sun FY, Atkins KE, Bosse NI, Clifford S, Russell TW, Deol AK, Liu Y, Procter SR, Leclerc QJ, Medley G, Knight G, Munday JD, Kucharski AJ, Pearson CAB, Klepac P, Prem K, Houben RMGJ, Endo A, Flasche S, Davies NG, Diamond C, van Zandvoort K, Funk S, Auzenbergs M, Rees EM, Tully DC, Emery JC, Quilty BJ, Abbott S, Villabona-Arenas CJ, Hué S, Hellewell J, Gimma A, Jarvis CI (2020). Global, regional, and national estimates of the population at increased risk of severe COVID-19 due to underlying health conditions in 2020: a modelling study. Lancet Glob Health.

[19] Winter SM (2020) Pandemic stress scale. Charité Universitätsmedizin Berlin, Berlin, Germany

[20] Berry JO, Jones WH (1995). The parental stress scale: Initial psychometric evidence. J Soc Pers Rel.

[21] Löwe B, Wahl I, Rose M, Spitzer C, Glaesmer H, Wingenfeld K, Schneider A, Brähler E (2010). A 4-item measure of depression and anxiety: Validation and standardization of the Patient Health Questionnaire-4 (PHQ-4) in the general population. J Affect Disord.

[22] Benyamini Y, Leventhal EA, Leventhal H (1999). Self-assessments of health: What do people know that predicts their mortality?. Res Aging.

[23] Runyan DK, Cox CE, Dubowitz H, Newton RR, Upadhyaya M, Kotch JB, Leeb RT, Everson MD, Knight ED (2005). Describing maltreatment: Do child protective service reports and research definitions agree?. Child Abuse Negl.

[24] Felitti VJ, Anda RF, Nordenberg D, Williamson DF, Spitz AM, Edwards V, Marks JS (1998). Relationship of childhood abuse and household dysfunction to many of the leading causes of death in adults: The Adverse Childhood Experiences (ACE) Study. Am Journal Prev Med.

[25] Teicher MH, Parigger A (2015). The ‘Maltreatment and Abuse Chronology of Exposure’(MACE) scale for the retrospective assessment of abuse and neglect during development. PLoS ONE.

[26] Hecker T, Boettcher VS, Landolt MA, Hermenau K (2019). Child neglect and its relation to emotional and behavioral problems: A cross-sectional study of primary school-aged children in Tanzania. Dev Psychopathol.

[27] Isele D, Teicher MH, Ruf-Leuschner M, Elbert T, Kolassa I-T, Schury K, Schauer M (2014). KERF–ein Instrument zur umfassenden Ermittlung belastender Kindheitserfahrungen. Z Klin Psychol und Psychother.

[28] Barnett D (1994). The impact of subtype, frequency, chronicity, and severity of child maltreatment on social competence and behavior problems. Dev Psychopathol.

[29] Destatis DB (2020) Mikrozensus. https://www-genesis.destatis.de/genesis/online?operation=statistic&levelindex=0&levelid=1605081838274&code=12211-abreadcrumb. Accessed 9 Nov 2020

[30] Stadelmann S, Perren S, Kölch M, Groeben M, Schmid M (2010). Psychisch kranke und unbelastete Eltern. Kindheit und Entwicklung.

[31] Kölch M, Schmid M (2008). Elterliche Belastung und Einstellungen zur Jugendhilfe bei psychisch kranken Eltern: Auswirkungen auf die Inanspruchnahme von Hilfen. Prax Kinderpsychol Kinderpsychiatr.

[32] Witt A, Brown RC, Plener PL, Brähler E, Fegert JM (2017). Child maltreatment in Germany: prevalence rates in the general population. Child Adolescent Psychiatry Ment Health.

[33] Patrick SW, Henkhaus LE, Zickafoose JS, Lovell K, Halvorson A, Loch S, Letterie M, Davis MM (2020). Well-being of parents and children during the COVID-19 pandemic: a national survey. Pediatrics.

[34] Sidebotham P, Golding J, Team AS (2001). Child maltreatment in the “Children of the Nineties”: a longitudinal study of parental risk factors. Child Abuse Negl.

[35] Spinelli M, Lionetti F, Setti A, Fasolo M (2020). Parenting stress during the COVID-19 outbreak: socioeconomic and environmental risk factors and implications for children emotion regulation. Fam Process.

[36] Fancourt D, Steptoe A, Bu F (2020). Trajectories of depression and anxiety during enforced isolation due to COVID-19: longitudinal analyses of 59,318 adults in the UK with and without diagnosed mental illness. medRxiv.

[37] Middelburg RA, Rosendaal FR (2020). COVID-19: how to make between-country comparisons. Int J Infect Dis.

[38] Dvorsky MR, Breaux R, Becker SP (2020). Finding ordinary magic in extraordinary times: child and adolescent resilience during the COVID-19 pandemic. Eur Child Adolesc Psychiatry.

[39] Bundesministerium für Familie, Frauen und Jugend (2017) Gelebte Vielfalt: Familien mit Migrationshintergrund in Deutschland. https://www.bmfsfj.de/blob/116880/83c02ec19dbea15014d7868048f697f2/gelebte-vielfalt--familien-mit-migrationshintergrund-in-deutschland-data.pdf. Accessed 9 Nov 2020

