# Supplementary material for: Families in the COVID-19 pandemic: parental stress, parent mental health and the occurrence of adverse childhood experiences—results of a representative survey in Germany
Source: Eur Child Adolesc Psychiatry. 2021 Mar 1;31(7):1–13. doi: 10.1007/s00787-021-01739-0 (PMC7917379; doi:10.1007/s00787-021-01739-0)
Supplement: Supplementary file 1 — Supplementary file1 (PDF 174 KB) [file 787_2021_1739_MOESM1_ESM.pdf]

Article title: Families in the COVID-19 Pandemic: Parental Stress, Parent Mental Health and the Occurrence of Adverse Childhood Experiences: Results of a Representative Survey in Germany

Journal: European Child & Adolescent Psychiatry

Authors: Claudia Calvano PhD[1], Lara Engelke MSc[2], Jessica Di Bella PhD[1], Jana Kindermann MSc[1], Babette Renneberg PhD[2*], & Sibylle M. Winter MD[1*]

*shared senior authors

Affiliations: [1] Charité - Universitätsmedizin Berlin, Corporate Member of Freie Universität Berlin, Humboldt-Universität zu Berlin, Berlin Institute of Health (BIH)
Department of Child and Adolescent Psychiatry, Psychosomatics and Psychotherapy
[2] Freie Universitaet of Berlin, Department of Clinical Psychology and Psychotherapy

Corresponding author: Claudia Calvano, PhD**,** Email: claudia.calvano@charite.de

Pandemic Stress Scale[1]

Dear parents,

we would like to measure additional family burden during the Corona-Pandemic in the last months.

For this reason, we ask you to answer the following questions.

	yes	no
Was a family member infected with the coronavirus?	○	○
Did a family member have to go to the hospital due to a corona infection?	○	○
Has a family member died due to a corona infection?	○	○
Has short-time work been ordered for a family member?	○	○
Has a family member lost their job due to the pandemic?	○	○
Has a family member suffered significant financial loss due to the pandemic?	○	○

[1] Winter SM (2020) Pandemic Stress Scale. Charité Universitätsmedizin Berlin, Berlin, Germany

Since the beginning of the Corona-Pandemic, which month was the one with the highest burden?

(multiple choice is possible)

- o February 2020
- o March 2020
- o April 2020
- o May 2020
- o June 2020
- o July 2020
- o All months were equally stressful.
- o No month was stressful.

Please answer the following questions on a scale from 1 ("not at all stressful") to 5 ("extremely stressful"):

Please relate your answers to the point in time with the greatest burden since the beginning of the Corona-Pandemic.

	**not at all stressful**				**extremely stressful**	**not applicable**
How stressful were the restrictions overall for you?	○	○	○	○	○	○
How stressful was the daycare closing for you?	○	○	○	○	○	○
How stressful was the school closing for you?	○	○	○	○	○	○
How stressful was doing the homework for you online?	○	○	○	○	○	○
How stressful was the closure of your company for you?	○	○	○	○	○	○
How stressful was the home office for you?	○	○	○	○	○	○
How stressful was maintaining the daily structure?	○	○	○	○	○	○
How stressful was the social distance to your family and friends for you?	○	○	○	○	○	○
How stressful was the restriction of outside activities?	○	○	○	○	○	○

How stressful was worrying about your health?	○	○	○	○	○	○
How stressful was worrying about the health of others?	○	○	○	○	○	○
How stressful were the restrictions on you considering medical care?	○	○	○	○	○	○
How stressful were the restrictions on you considering psychotherapeutic / psychiatric care?	○	○	○	○	○	○
How stressful were the restrictions on you considering youth welfare?	○	○	○	○	○	○
