# Supplementary material for: Families in the COVID-19 pandemic: parental stress, parent mental health and the occurrence of adverse childhood experiences—results of a representative survey in Germany
Source: Eur Child Adolesc Psychiatry. 2021 Mar 1;31(7):1–13. doi: 10.1007/s00787-021-01739-0 (PMC7917379; doi:10.1007/s00787-021-01739-0)
Supplement: Supplementary file 2 — Supplementary file2 (PDF 107 KB) [file 787_2021_1739_MOESM2_ESM.pdf]

Article title: Families in the COVID-19 Pandemic: Parental Stress, Parent Mental Health and the Occurrence of Adverse Childhood Experiences: Results of a Representative Survey in Germany

Journal: European Child & Adolescent Psychiatry

Authors: Claudia Calvano PhD[1], Lara Engelke MSc[2], Jessica Di Bella PhD[1], Jana Kindermann MSc[1], Babette Renneberg PhD[2*], & Sibylle M. Winter MD[1*]

*shared senior authors

Affiliations: [1] Charité - Universitätsmedizin Berlin, Corporate Member of Freie Universität Berlin, Humboldt-Universität zu Berlin, Berlin Institute of Health (BIH), Department of Child and Adolescent Psychiatry, Psychosomatics and Psychotherapy

[2] Freie Universitaet of Berlin, Department of Clinical Psychology and Psychotherapy

Corresponding author: Claudia Calvano, PhD**,** Email: claudia.calvano@charite.de

Supplementary Material 2: Assessment of Adverse Childhood Experiences

A modified and shortened version of the pediMACE [1, 2] was applied. All items referred to actions either by the parents only (supervisory neglect, emotional neglect) or by the parents or other adults in the household (verbal emotional abuse, nonverbal emotional abuse, physical abuse, physical neglect, witnessing domestic violence), except the item on sexual abuse, which referred to any adult or clearly older person [2].

Instruction:

The following questions relate to the topic of domestic violence.

Parenthood is not always easy, as life circumstances are stressful and children can be challenging. We also know that such situations can affect education and education not always runs perfectly.

Since you have the best overview, we ask for your answers here.

It is important that you answer the questions as honestly as possible.

In the following, some questions are asked in the yes / no format. The questions initially relate to the entire life span of the child/ children in your household. If you answer "yes" to a question, then

the question appears again on the next page and asks how often this event has occurred since the beginning of the corona pandemic.

All information in this survey is anonymous.

Families in the COVID-19 pandemic – Supplementary Material 2: Assessment of Adverse Childhood Experiences

Item 1 as example for the stepwise assessment:

1. Were there ever severe stressful living conditions (e.g. violence, neglect, abuse) for the child/children?

- o Yes
- o No

If yes:

1.1 How often have there been severe stressful living conditions (violence, neglect, abuse) for the child/ children in your household, since the beginning of the corona pandemic?

Compared to January 2020 ...

- o significantly more often
- o a little more often
- o comparably often
- o a little more seldom
- o significantly more seldom

Please see below for the specific ACE subtypes and related item wordings as used in our study.

Item #	ACE Subtype	Wording
2	Verbal emotional abuse	Has at least one adult who lives in the household with the child/ children ever yelled at the child/ children more than a few times a year?<br><br>How often has/ have the child/ children been yelled at by an adult in your household, since the beginning of the corona pandemic?
3	Nonverbal emotional abuse	Has there ever been a situation where the child/ children in your household had to take over a responsibility, which should only be taken over by a significantly older child or adult?<br><br>How often did the child/ children in your household have to take over a responsibility, which should only be taken over by a significantly older child or adult, since the beginning of the corona pandemic?
4	Emotional neglect	Was there ever a time when neither the father nor the mother (or other main caregivers / parent-like persons) tried to understand the feelings of the child /children or could be there for them?

		How often have neither the father nor the mother (or other main caregivers / parental-like persons) been able to understand the feelings of the child /children or could be there for them, since the beginning of the corona pandemic?
5	Physical abuse	Did at least one adult living in the household with the child/ children ever intentionally pushed, pinched, slapped or hit the child/ children with the fist or kicked the child/ children with the foot?
		How often have there been situations in which at least one adult who lives in the household with the child/ children intentionally pushed, pinched, slapped or hit the child/ children with the fist or kicked the child/ children with the foot, since the beginning of the corona pandemic?
6	Supervisory neglect	Has there ever been a time when at least one parent paid too little attention or did not protect the child/ children?
		How often have there been situations in which at least one parent paid too little attention or did not protect the child/ children, since the beginning of the corona pandemic?
7	Physical neglect	Has there ever been a time when the child/ children in your household did not have enough to eat?
		How often have there been situations in which the child/children in your household did not have enough to eat, since the beginning of the corona pandemic?
8	Witnessing domestic violence	Sometimes parents or other adults (new partners, grandparents) with whom the child/ children live together fight.
		Has/have your child/children ever seen adults who live with the child/ children fight violently?
		How often have there been situations in which your child/ children experienced how adults who lived with the child/children argued violently, since the beginning of the corona pandemic?

9	Sexual abuse	The following question relates to adults who live in the household and other adults or others who did not live with your child/ children at home. Please indicate whether your child/ children did/ does experience the following situation.<br><br>Has it ever happened that an adult touched your child's/ children's intimate parts of the body without the child/ children wanting this? Or did it happen that your child/ children was/ were forced to touch another person's body?<br><br>How often have there been situations in which an adult person has touched your child's/ children's intimate parts of the body without the child/ children wanting this or forced the child/ children to touch another person's body, since the beginning of the corona pandemic?
10	Alcohol or substance abuse in the household	Has/ have your child/ children ever lived with someone who has alcohol problems or is addicted to alcohol or has used drugs?<br><br>How often have domestic problems occurred in connection with the alcohol or drug problem, since the beginning of the corona pandemic?
11	Mental illness in the household	Has/have your child/ children ever lived with someone who was depressed or mentally ill?<br><br>How often have there been domestic difficulties related to depression or mental illness, since the beginning of the corona pandemic?

References Supplementary Material 2:

1. Hecker T, Boettcher VS, Landolt MA, Hermenau K (2019) Child neglect and its relation to emotional and behavioral problems: A cross-sectional study of primary school-aged children in Tanzania. Dev Psychopathol 31 (1):325-339
2. Isele D, Teicher MH, Ruf-Leuschner M, Elbert T, Kolassa I-T, Schury K, Schauer M (2014) KERF–ein Instrument zur umfassenden Ermittlung belastender Kindheitserfahrungen. Z Klin Psychol Psychother 43 (2): 121–130.
