# Supplementary material for: Families in the COVID-19 pandemic: parental stress, parent mental health and the occurrence of adverse childhood experiences—results of a representative survey in Germany
Source: Eur Child Adolesc Psychiatry. 2021 Mar 1;31(7):1–13. doi: 10.1007/s00787-021-01739-0 (PMC7917379; doi:10.1007/s00787-021-01739-0)
Supplement: Supplementary file 3 — Supplementary file3 (PDF 102 KB) [file 787_2021_1739_MOESM3_ESM.pdf]

Article title: Families in the COVID-19 Pandemic: Parental Stress, Parent Mental Health and the Occurrence of Adverse Childhood Experiences: Results of a Representative Survey in Germany

Journal: European Child & Adolescent Psychiatry

Authors: Claudia Calvano PhD[1], Lara Engelke MSc[2], Jessica Di Bella PhD[1], Jana Kindermann MSc[1], Babette Renneberg PhD[2*], & Sibylle M. Winter MD[1*]

*shared senior authors

Affiliations: [1] Charité - Universitätsmedizin Berlin, Corporate Member of Freie Universität Berlin, Humboldt-Universität zu Berlin, Berlin Institute of Health (BIH)

Department of Child and Adolescent Psychiatry, Psychosomatics and Psychotherapy

[2] Freie Universitaet of Berlin, Department of Clinical Psychology and Psychotherapy

Corresponding author: Claudia Calvano, PhD**,** Email: claudia.calvano@charite.de

Table S3

Bivariate correlations between the specific areas of pandemic-related stress and the outcomes on parent level

	overall burden	childcare closures	school closures	home-work online	work-place closures	home office	maintain daily structure	social distancing	restrict. outside activ.	worries own health	worries others' health	restrict medical treatment	restrict psychoth. treatment	restrict child services
PS	.229***	.235***	.284***	.338***	.138*	.251***	.314***	.104**	.153***	.162***	.090**	.177***	.289***	.393***
GS	.300***	.089*	.290***	.317***	.261***	.292***	.362***	.247***	232***	.323***	.299***	.297***	.339***	.298***
SH	-.137***	.002	-.150***	-.191***	-.102	-.199***	-.153***	-.077*	-.097**	-.293***	-.201***	-.236***	-.273***	-.265***
ANX	.240***	.107*	.205***	.182**	.236***	.136**	.185***	.187***	.158***	.283***	.263***	.240***	.288***	.302***
DEPR	.244***	.134**	.206***	.203***	.113*	.141**	.258***	.191***	.185***	.220***	.213***	.196***	.246***	.223***

Notes. PS = Parental stress; GS = general stress; SH = subjective health rating; ANX= anxiety; DEPR = Depression; for the subjective health rating, higher scores indicate better health, for the other outcomes, higher scores indicate higher burden, stress and symptoms; all parental outcomes with respect to the time of the subjectively highest burden; * p < .05. ** p < .01. *** p < .001.
