# Supplementary material for: Families in the COVID-19 pandemic: parental stress, parent mental health and the occurrence of adverse childhood experiences—results of a representative survey in Germany
Source: Eur Child Adolesc Psychiatry. 2021 Mar 1;31(7):1–13. doi: 10.1007/s00787-021-01739-0 (PMC7917379; doi:10.1007/s00787-021-01739-0)
Supplement: Supplementary file 4 — Supplementary file4 (PDF 125 KB) [file 787_2021_1739_MOESM4_ESM.pdf]

Article title: Families in the COVID-19 Pandemic: Parental Stress, Parent Mental Health and the Occurrence of Adverse Childhood Experiences: Results of a Representative Survey in Germany

Journal: European Child & Adolescent Psychiatry

Authors: Claudia Calvano PhD[1], Lara Engelke MSc[2], Jessica Di Bella PhD[1], Jana Kindermann MSc[1], Babette Renneberg PhD[2*], & Sibylle M. Winter MD[1*]

*shared senior authors

Affiliations: [1] Charité - Universitätsmedizin Berlin, Corporate Member of Freie Universität Berlin, Humboldt-Universität zu Berlin, Berlin Institute of Health (BIH)

Department of Child and Adolescent Psychiatry, Psychosomatics and Psychotherapy

[2] Freie Universitaet of Berlin, Department of Clinical Psychology and Psychotherapy

Corresponding author: Claudia Calvano, PhD**,** Email: claudia.calvano@charite.de

Supplementary Material 4

Table S4

Group differences in change of ACEs on parent outcomes

	Increase	Pandemic-related stress		Parental stress		General stress		Subjective health		Anxiety		Depression	
		M (SD)	d	M (SD)	d	M (SD)	d	M (SD)	d	M (SD)	d	M (SD)	d
WDV	no (n=236)	31.98 (9.12)	0.46***	37.64 (9.35)	0.72***	6.33 (4.29)	0.66***	6.59 (2.13)	0.50***	1.11 (1.09)	0.46***	1.38 (1.18)	0.52***
	yes (n=97)	36.41 (10.65)		44.51 (10.03)		9.03 (3.62)		5.53 (2.14)		1.64 (1.31)		1.98 (1.13)	
VEA	no (n=192)	31.16 (9.79)	0.45***	37.22 (9.03)	0.88***	5.46 (3.91)	0.54***	6.76 (2.03)	0.35**	1.09 (1.22)	0.17	1.27 (1.21)	0.40***
	yes (n=140)	35.69 (10.48)		45.22 (9.12)		7.66 (4.23)		5.99 (2.43)		1.29 (1.16)		1.77 (1.34)	
NEA	no (n=37)	34.30 (12.99)	0.32	35.05 (9.25)	0.88***	6.93 (4.78)	0.31	6.13 (2.24)	0.16	1.25 (1.39)	0.33	1.58 (1.20)	0.25
	yes (n=45)	37.91 (9.45)		45.17 (11.12)		8.26 (3.98)		5.74 (2.53)		1.68 (1.20)		1.86 (1.05)	
EN	no (n=94)	35.81 (9.90)	0.30	39.00 (11.60)	0.35*	6.35 (4.51)	0.24	6.43 (2.22)	0.16	1.20 (1.13)	0.14	1.39 (1.17)	0.24
	yes (n=65)	39.14 (12.47)		43.26 (12.88)		7.46 (4.93)		6.04 (2.70)		1.37 (1.40)		1.68 (1.32)	
PA	no (n=40)	30.87 (8.19)	0.63*	44.49 (10.05)	1.04***	7.47 (3.67)	0.46	5.67 (2.22)	0.34	1.36 (1.10)	0.64*	1.81 (0.97)	0.39
	yes(n=17)	37.00 (12.68)		54.21 (7.44)		9.42 (5.41)		4.89 (2.40)		2.11 (1.32)		2.22 (1.21)	

	Increase	Pandemic-related stress		Parental stress		General stress		Subjective health		Anxiety		Depression	
		M (SD)	d	M (SD)	d	M (SD)	d	M (SD)	d	M (SD)	d	M (SD)	d
SN	no (n=57)	34.79 (9.27)	0.45*	41.08 (11.23)	0.09	7.35 (4.39)	0.30	6.35 (2.58)	0.36	1.21 (1.08)	0.31	1.75 (1.30)	0.01
	yes (n=39)	39.14 (10.13)		42.00 (9.08)		8.81 (5.39)		5.45 (2.30)		1.59 (1.41)		1.75 (1.15)	
MI	no (n=101)	31.91 (8.95)	0.81***	38.52 (9.64)	1.04***	7.79 (3.96)	0.65***	5.79 (2.21)	0.61**	1.41 (1.12)	0.99***	1.84 (1.21)	0.54**
	yes (n=41)	39.78 (11.33)		47.67 (10.06)		10.35 (3.82)		4.37 (2.57)		2.53 (1.14)		2.50 (1.22)	
AS	no (n=31)	37.23 (10.88)	0.59	43.12 (10.56)	0.89*	8.15 (4.35)	0.45	6.32 (2.36)	0.64	1.44 (1.24)	1.42**	1.69 (1.20)	0.74
	yes (n=6)	31.00 (7.96)		52.86 (13.33)		10.00 (2.45)		4.86 (1.68)		3.14 (0.90)		2.57 (1.13)	
SEV	no (n=43)	35.49 (10.60)	0.44	42.58 (11.48)	0.64*	8.38 (4.07)	0.11	6.18 (2.10)	0.48	1.45 (1.17)	0.66*	1.69 (1.22)	0.28
	yes n=(23)	40. 35 (11.85)		49.54 (9.80)		8.85 (4.99)		5.08 (2.67)		2.28 (1.43)		2.04 (1.31)	

Notes. WDV = witnessing domestic violence, VEA = verbal emotional abuse, NEA = nonverbal emotional abuse, EN = emotional neglect, PA = physical abuse, SN = supervisory neglect, MI = mental illness in the household, AS = alcohol or substance abuse in the household; SEV = severe forms of ACEs like maltreatment, neglect, violence; d = Cohen's d for effect size (d=0.2 small, d = 0.5 medium, d =.08 large); * p < .05, ** p < .01, *** p = .001.
