# Supplementary material for: Families in the COVID-19 pandemic: parental stress, parent mental health and the occurrence of adverse childhood experiences—results of a representative survey in Germany
Source: Eur Child Adolesc Psychiatry. 2021 Mar 1;31(7):1–13. doi: 10.1007/s00787-021-01739-0 (PMC7917379; doi:10.1007/s00787-021-01739-0)
Supplement: Supplementary file 5 — Supplementary file5 (PDF 121 KB) [file 787_2021_1739_MOESM5_ESM.pdf]

Article title: Families in the COVID-19 Pandemic: Parental Stress, Parent Mental Health and the Occurrence of Adverse Childhood Experiences: Results of a Representative Survey in Germany

Journal: European Child & Adolescent Psychiatry

Authors: Claudia Calvano PhD[1], Lara Engelke MSc[2], Jessica Di Bella PhD[1], Jana Kindermann MSc[1], Babette Renneberg PhD[2*], & Sibylle M. Winter MD[1*]
*shared senior authors

Affiliations: [1] Charité - Universitätsmedizin Berlin, Corporate Member of Freie Universität Berlin, Humboldt-Universität zu Berlin, Berlin Institute of Health (BIH)
Department of Child and Adolescent Psychiatry, Psychosomatics and Psychotherapy
[2] Freie Universitaet of Berlin, Department of Clinical Psychology and Psychotherapy

Corresponding author: Claudia Calvano, PhD**,** Email: claudia.calvano@charite.de

Supplementary Material 5

Factors associated with an increase in ACEs

The following risk factors were tested for an increase vs. decrease/no change in witnessing domestic violence (n=97 increase) and verbal emotional abuse (n=140 increase): sociodemographic factors (parent age, parent sex, number of children, average child age, single parenting, SES index), parent-related risk factors (parental alcohol abuse, parent mental disorder, parental history of physical or sexual abuse in childhood or adolescence, parental experience of physical or sexual violence in adulthood), and pandemic-related risk factors (parent among the risk group for COVID-19, short-time work, loss of job, severe financial loss). Results for parent age, child age and the number of children in the household are summarized in Table S5a, results for categorial risk factors are summarized in Table S5b.

Table S5a

Differences in parent and child age for witnessing domestic violence and verbal emotional abuse

	Witnessing domestic violence			Verbal emotional abuse		
	decrease/no change<br>n=236<br>M (SD)	increase<br>n=97<br>M (SD)	p	decrease / no change<br>n=192<br>M (SD)	increase<br>n=140<br>M (SD)	p
Parent age	41.70 (7.91)	39.69 (7.26)	.032	42.33 (7.71)	39.62 (7.00)	.001
Child age	10.18 (4.88)	8.10 (4.54)	<.001	10.17 (4.75)	7.82 (4.26)	<.001
Number of children	1.73 (0.83)	1.76 (0.75)	.763	1.83 (0.80)	1.86 (0.72)	.719

Table S5b

Differences in categorial risk factors for witnesses of domestic violence and verbal emotional abuse

		witnessing domestic violence				verbal emotional abuse	
		increase	p			increase	p
**Sociodemographic risk factors**				**Sociodemographic risk factors**			
Parent sex	mothers (n=192)	26.0%	.176	Parent sex	mothers (n=184)	41.3%	.722
	fathers (n=140)	32.9%			fathers (n=148)	43.2%	
Single parenting	yes (n=46)	19.6%	.855	Single parenting	yes (n=43)	51.2%	.361
	no (n=9)	22.2%			no (n=4)	75.0%	
SES[a]	low (n=38)	15.8%	.121	SES[a]	low (n=28)	28.6%	.048
	medium (n=193)	32.1%			medium (n=185)	48.1%	
	high (n=101)	27.7%			high (n=119)	37.0%	
**Pandemic-specific risk factors**				**Pandemic-specific risk factors**			
Job loss	yes (n=31)	48.4%	.013	Job loss	yes (n=29)	62.1%	.024
	no (n=302)	27.2%			no (n=304)	40.5%	
Financial loss	yes (n=101)	32.7%	.360	Financial loss	yes (n=83)	53.0%	.021
	no (n=231)	27.7%			no (n=249)	38.6%	

		witnessing domestic violence				verbal emotional abuse	
		increase	p			increase	p
Reduced working hours	yes (n=107)	32.7%	.322	Reduced working hours	yes (n=109)	45.9%	.339
	no (n=226)	27.4%			no (n=223)	40.4%	
Parent among COVID-19 risk group[b]	yes (n=47)	34.0%	.733	Parent among COVID-19 risk group[b]	yes (n=38)	47.4%	.378
	no (n=13)	38.5%			no (n=13)	61.5%	
**Parent-related risk factors**				**Parent-related risk factors**			
Parental history of child abuse	yes (n=111)	37.8%	.014	Parental history of child abuse	yes (n=106)	49.1%	.082
	no (n=221)	24.9%			no (n=226)	38.9%	
Parental Experience of violence	yes (n=58)	31.0%	.707	Parental experience of violence	yes (n=52)	61.5%	.002
	no (n=273)	28.6%			no (n=279)	38.7%	
Parental mental disorder	yes (n=61)	36.1%	.193	Parental mental disorder	yes (n=50)	54.0%	.066
	no (n=271)	27.7%			no (n=282)	40.1%	
Parental risk for alcohol abuse[c]	yes (n=28)	35.7%	.429	Parental risk for alcohol abuse[c]	yes (n=22)	45.5%	.756
	no (n=304)	28.6%			no (n=309)	42.1%	

Notes. [a]according to Winkler-Index [1]; risk group of severe COVID-19 according to Clark et al. [2]; parental risk for alcohol abuse according to PHQ-D [3].

References Supplementary Material 5

1. Lampert T, Müters S, Stolzenberg H, Kroll LE, Group KS (2014) Messung des sozioökonomischen Status in der KiGGS-Studie. Bundesgesundheitsblatt-Gesundheitsforschung-Gesundheitsschutz 57 (7):762-770

2. Löwe B, Spitzer RL, Zipfel S, Herzog W (2002) PHQ-D: Gesundheitsfragebogen für Patienten; Manual Komplettversion und Kurzform. Pfizer GmbH

3. Clark A, Jit M, Warren-Gash C, Guthrie B, Wang HHX, Mercer SW, Sanderson C, McKee M, Troeger C, Ong KL, Checchi F, Perel P, Joseph S, Gibbs HP, Banerjee A, Eggo RM, Nightingale ES, O'Reilly K, Jombart T, Edmunds WJ, Rosello A, Sun FY, Atkins KE, Bosse NI, Clifford S, Russell TW, Deol AK, Liu Y, Procter SR, Leclerc QJ, Medley G, Knight G, Munday JD, Kucharski AJ, Pearson CAB, Klepac P, Prem K, Houben RMGJ, Endo A, Flasche S, Davies NG, Diamond C, van Zandvoort K, Funk S, Auzenbergs M, Rees EM, Tully DC, Emery JC, Quilty BJ, Abbott S, Villabona-Arenas CJ, Hué S, Hellewell J, Gimma A, Jarvis CI (2020) Global, regional, and national estimates of the population at

increased risk of severe COVID-19 due to underlying health conditions in 2020: a modelling study. Lancet Glob Health 8 (8):e1003-e1017. doi:https://doi.org/10.1016/S2214-109X(20)30264-3
